# The role of dual energy computed tomography in the differentiation of acute gout flares and acute calcium pyrophosphate crystal arthritis

**DOI:** 10.1007/s10067-021-05949-4

**Published:** 2021-10-09

**Authors:** Dmitrij Kravchenko, Pantelis Karakostas, Daniel Kuetting, Carsten Meyer, Peter Brossart, Charlotte Behning, Valentin S. Schäfer

**Affiliations:** 1 Department of Diagnostic and Interventional Radiology, University Hospital Bonn, Venusberg-Campus 1, 53127 Bonn, Germany; 2 Clinic of Internal Medicine III, Hematology, Oncology, Rheumatology and Clinical Immunology, University Hospital Bonn, Bonn, Germany; 3 Institute for Medical Biometrics, Informatics and Epidemiology (IMBIE), University Hospital Bonn, Bonn, Germany

**Keywords:** Acute CPP crystal arthritis, Diagnosis, Dual energy computed tomography, Gout, Ultrasound

## Abstract

**Objectives:**

To analyse the diagnostic impact of dual energy computed tomography (DECT) in acute gout flares and acute calcium pyrophosphate (CPP) crystal arthritis when compared to the gold standard of arthrocentesis with compensated polarised light microscopy. Microscopy results were also compared to musculoskeletal ultrasound (MUS), conventional radiographs, and the suspected clinical diagnosis (SCD).

**Methods:**

Thirty-six patients with a suspected gout flare (*n* = 24) or acute CPP crystal arthritis (*n* = 11, *n* = 1 suffered from neither) who received a DECT and underwent arthrocentesis were included. Two independent readers assessed DECT images for signs of monosodium urate crystals or calcium pyrophosphate deposition.

**Results:**

Sensitivity of DECT for gout was 63% (95% CI 0.41–0.81) with a specificity of 92% (0.41–0.81) while sensitivity and specificity for acute CPP arthritis were 55% (0.23–0.83) and 92% (0.74–0.99), respectively. MUS had the highest sensitivity of all imaging modalities with 92% (0.73–0.99) and a specificity of 83% (0.52–0.98) for gout, while sensitivity and specificity for acute CPP crystal arthritis were 91% (0.59–1.00) and 92% (0.74–0.99), respectively.

**Conclusion:**

DECT is an adequate non-invasive diagnostic tool for acute gout flares but might have a lower sensitivity than described by previous studies. Both MUS and SCD had higher sensitivities than DECT for acute gout flares and acute CPP crystal arthritis.

**Supplementary Information:**

The online version contains supplementary material available at 10.1007/s10067-021-05949-4.

**Table:** 

**Key Points** • *DECT offers a lower sensitivity for acute gout flares than previously described.* • *DECT sensitivity for acute CPP crystal arthritis is less than the already validated ultrasound.*	

## Introduction

The differentiation between acute gout flares and calcium pyrophosphate deposition disease (CPPD), more specifically acute CPP crystal arthritis, can sometimes prove to be difficult as patients often present with similar signs and symptoms [1]. The latest European League Against Rheumatism (EULAR) guidelines recommend synovial fluid or tophus aspiration with subsequent compensated polarised light microscopy (CPLM) in every suspected gout case. When aspiration is not possible, a clinical diagnosis of gout can be made if certain features such as monoarticular foot joint involvement, similar previous episodes, or rapid onset of symptoms are present. In recent years, novel diagnostic approaches such as non-invasive dual energy computed tomography (DECT) and established diagnostic tools such as musculoskeletal ultrasound (MUS) have been validated for diagnosing gout [2]. EULAR recommendations list MUS and DECT as grade A recommendations with a level 1b of evidence suggesting these non-invasive tools can aid diagnosis when CPLM does not provide diagnostic clues [2]. Such evidence is still lacking in regard to the value of DECT in CPP crystal arthritis.

Gout is caused by precipitation of monosodium urate (MSU) crystals in joints and soft tissues resulting in an inflammatory cascade [3]. MSU crystal deposition is facilitated by either an overproduction or, more commonly, an underexcretion. Acute gout flares are thought to be provoked by specific triggers such as alcohol excess or trauma. Genetic dispositions also play a role in the development of gout [4]. The Gout, Hyperuricemia and Crystal-Associated Disease Network (G-CAN) has published recommendation to differentiate gout states clarifying terms such as for example, urate (circulating form of the molecule generated by xanthine oxidase), gout flare (acute inflammation facilitated by MSU crystals), or tophus (congregation of MSU crystals and host response tissue forming a delineated structure in soft tissues) [5].

CPPD is thought to occur when an imbalance of inorganic pyrophosphate production and pyrophosphatase causes saturation and precipitation of CPP crystals inside of cartilage [6, 7]. EULAR has also published a recommendation for the standardisation of the nomenclature used in CPPD which differentiates terms such as CPPD as the umbrella term for any instance of CPP crystal occurrence, cartilage calcification as an isolate imaging finding, asymptomatic CPPD without clinical significance, osteoarthritis with CPPD, acute CPP crystal arthritis replacing the term “pseudogout”, and chronic CPP crystal arthritis [8].

Gout and CPPD can be differentiated based on their visual characteristics under CPLM: MSU crystals present as needle-shaped negatively birefringent crystals while CPP crystals are typically rhomboid-shaped and positively birefringent.

DECT might be able to offer similar insights as CPLM without its invasiveness and provide more information than MUS [9–11]. DECT functions by producing X-ray beams at two different energies measured in peak kilovoltage (kVp). Photons with dissimilar energies interact differently with certain materials based on their atomic number [12]. This allows DECT scans to colour code MSU crystals based on their Hounsfield units by utilizing a decomposition algorithm to analyse the two energy datasets. In principle, the same process can be applied to CPPD, with some minor caveats, theoreticaly aiding in the diagnosis of CPPD.

This is the first study on the diagnostic value of DECT in patients with acute arthritis due to gout or CPPD compared to arthrocentesis with CPLM, the suspected clinical diagnosis (SCD), and other commonly applied imaging modalities like MUS and conventional radiographs (CR). Furthermore, standard laboratory parameters were analysed using descriptive statistics.

## Methods

DECT images of 36 patients from the Department of Rheumatology and Clinical Immunology at the University Hospital Bonn, Germany, who were initially suspected of suffering from gout or CPPD of any joint and underwent arthrocentesis between May 31st 2018 and July 29th 2021, were retrospectively analysed. If patients underwent musculoskeletal ultrasound or received radiographs, these were also analysed. The ethics committee of the University Hospital Bonn (Ethikkommission an der Medizinischen Fakultät der Friedrich-Wilhelms-Universität Bonn) approved the study design and information processing (approval number Lfd.Nr.469/19). Due to the retrospective design, no patient consent was obtained.

### Inclusion/exclusion criteria

Inclusion criteria were age ≥ 18 years, patients with clinically suspected gout or CPPD who underwent DECT and CPLM. Exclusion criteria: patients who only underwent DECT or CPLM but not both.

### Dual energy computed tomography protocol

All DECT scans were performed with a SOMATOM® Force (Siemens Medical Solutions, Munich, Germany) dual source/dual detector scanner. Post-processing was done using the Syngo.via software suit from Siemens (VB20A, Munich, Germany) using a Siemens pre-set for gout. Detailed scan parameters are summarised in Supplementary Table S1. Non-enhanced images of the affected joint were obtained at 80 kV and at 150 kV with variable dose modulation at 0.75-mm slice thickness.

### Dual energy computed tomography evaluation

Two independent readers (CM and DK with respectively 4 and 3 years of DECT experience), blinded to the initial DECT reports, assessed the composite, 80 kV, 150 kV, and colour-coded datasets for signs of gout or CPP depositions using a standard workstation. The readers knew the suspected diagnosis based on the DECT request form, as they would in a typical clinical setting but were unaware of the CPLM results. MSU quantification was performed automatically by the software. Signs of gout were defined as demarcated soft tissue tophi, articular and juxta-articular osseous erosions, or green voxels in the colour-coded data set [9]. CPPD was defined as calcification in hyaline cartilage such as in the menisci or the triangular fibrocartilage complex on either conventional CT or colour-coded DECT images [13]. Both readers were aware of common DECT artefacts [14]. If the two readers differed in their conclusions, a third reader (DRLK, board certified radiologist with at least 4 years of DECT experience) was asked to read the images and serve as a tie breaker.

### Ultrasound examination

Ultrasound was performed by a board-certified musculoskeletal sonographer with DEGUM (German Society for Ultrasound in Medicine) level III (VSS), representing the highest certified level of ultrasound training in Germany, at the Department of Rheumatology using a GE Logiq S8 XDclear ultrasound machine, manufactured in 2018. A linear ultrasound probe with a range from 6 to 15 MHz and a hockey stick probe with a range from 8 to 18 MHz was applied. Ultrasound examinations were assessed for juxta-articular and soft tissue tophi, aggregates, erosions, and the double contour sign, as well as indirect signs such as hypervascularity and joint effusion of the most affected joint. Diagnostic criteria for gout included tophi or a positive double contour sign along the index joint [15]. CPPD diagnostic criteria included a thin hyperechoic band in hyaline cartilage, hyperechoic spots in cartilage, and intra-articular hyperechoic nodules [16]. Initial ultrasound examinations were not re-read.

### Conventional radiographs

Conventional radiographs were acquired under standard conditions utilizing Carestream DRX-Evolution machines (Carestream, Rochester, NY, USA). Images were screened for tophi and non-demineralizing erosive changes along articular or juxta-articular bone typical of changes in gout. Diagnostic criteria for CPPD included fine radiopaque densities in articular hyaline cartilage or fibrocartilage in typical locations such as the meniscus of the knee or the triangular fibrocartilage complex [17].

### Suspected clinical diagnosis

The suspected clinical diagnosis of the treating rheumatologist was based on the medical history (e.g. symptom onset) and clinical examination of the patient (e.g. podagra) at the Department of Rheumatology, University Hospital Bonn, Germany. Patients were additionally scored on the 2015 American College of Rheumatology (ACR)/EULAR gout classification criteria as previously described [18].

### Arthrocentesis

Arthrocentesis and synovial fluid analysis were performed by one of two experienced rheumatologists (VSS or PK) using an Olympus BX51-P polarizing microscope from 2016. CPLM was performed directly after arthrocentesis. The presence of negatively birefringent needle-shaped crystals was classified as gout while positively birefringent rhomboid-shaped crystals were classified as CPPD. Joint aspirate was analysed visually and sent for microbiological analysis to rule out septic arthritis.

### Laboratory parameters

Laboratory parameters were collected at the time of initial visit at the same time as MUS, radiographs, and CPLM. The most recent laboratory parameters associated with gout or CPPD were analysed. They included levels of serum urate (normal: 2.6–6.0 mg/dL), highest serum urate ever (2.6–6.0 mg/dL), calcium (2.2–2.55 mmol/l), magnesium (0.77–1.03 mmol/l), organic phosphate (0.81–1.45 mmol/l), iron (33–193 µg/dL), ferritin (30–400 ng/ml), CRP (< 3 mg/l), leukocytes (3.6–10.5 cells/µl), haemoglobin (13.5–17.2 g/dl), thrombocytes (150–370 G/l), neutrophils (42–77%), and TSH (0.27–4.2 µU/ml).

### Statistical analysis

Statistical analysis was performed using the R software environment (version 4.0.2) by a trained statistician (CB). Continuous variables are described as mean and standard deviation and as median and inter-quartile range (IQR) where normality could not be assumed. Categorical variables are presented as absolute and relative frequencies. Inter-reader agreement for DECT assessment was analysed by Krippendorff’s alpha using the R package irr [19]. Sensitivities and specificities are reported with 95% confidence intervals. The Student’s *t* test was used for parametric data, while the Mann-Whitney *U* test was used when normality could not be assumed. Welch’s test was used to compare non-binary lab parameters when no equal variance between the variables could be assumed. All relevant data is available from within the manuscript.

## Results

### Clinical characteristics

**Fig. 1:**
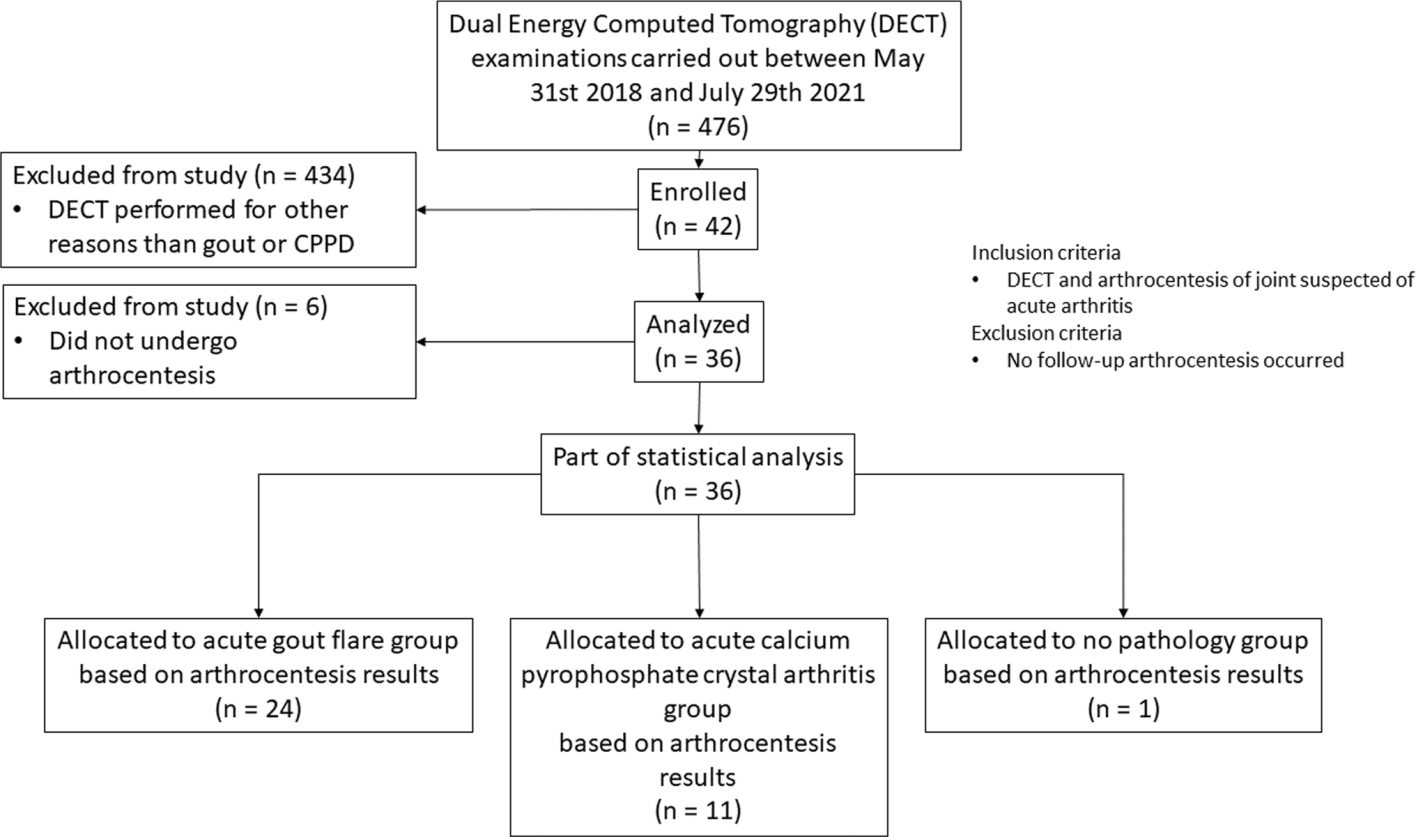
CONSORT (**Con**solidated **S**tandards **O**f **R**eporting **T**rials) diagram visualising patient group allocation based on arthrocentesis findings

**Fig. 2:**
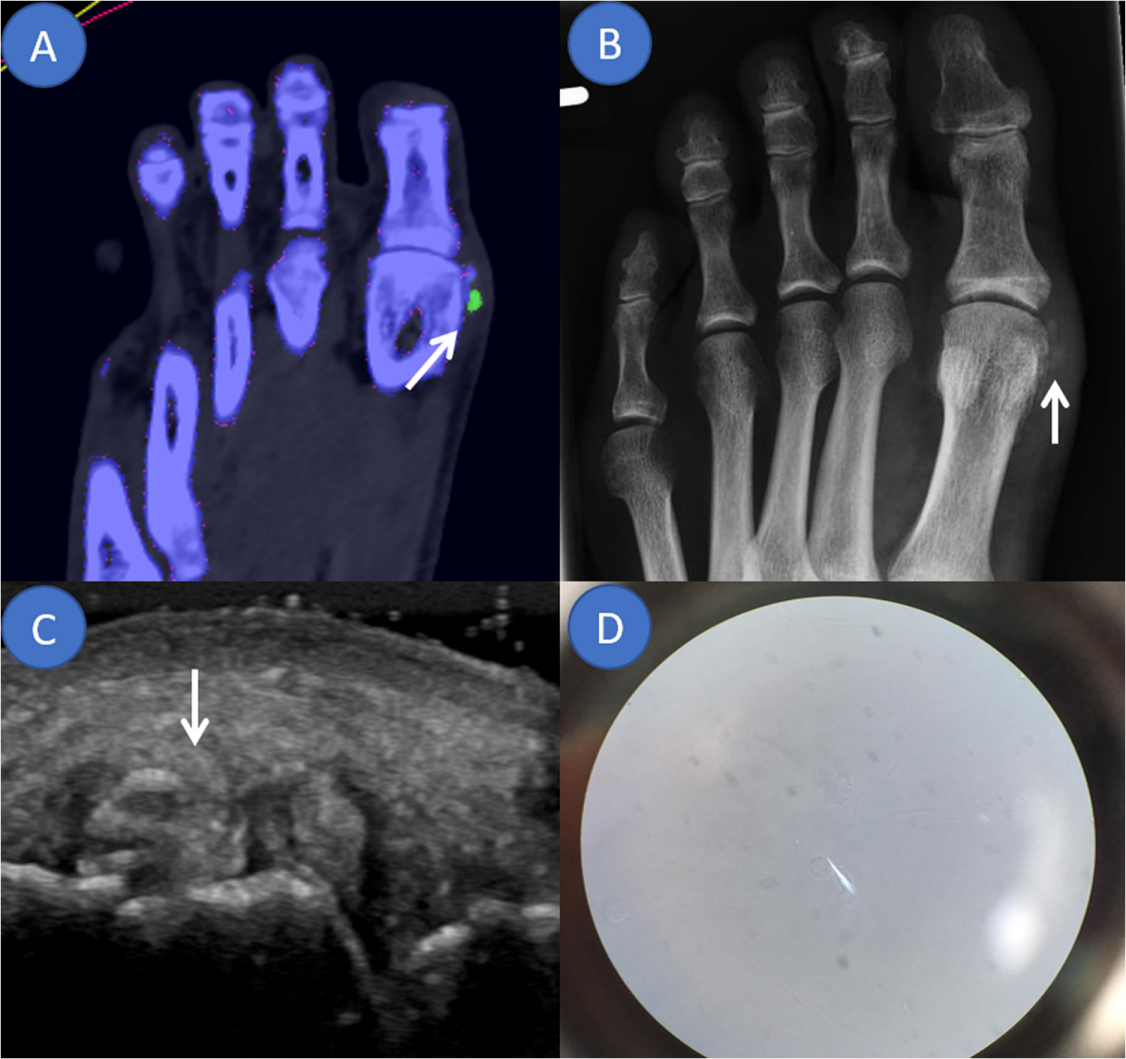
Overview of applied imaging modalities in an acute gout flare of the first metatarsophalangeal joint. The same mono sodium urate deposit marked by an arrow in different imaging modalities. **A** Dual energy computed tomography colour-coded image highlighting monosodium urate deposits in green. **B** Conventional radiograph demonstrating an irregular opacification along the medial aspect of the distal first metatarsal bone suggestive of gout. **C** Ultrasound showing a tophus and an erosion as well as aggregates. **D** Compensated polarised light microscopy of the aspirated synovial fluid confirming the diagnosis of gout by demonstrating spindle shaped deposits

**Fig. 3:**
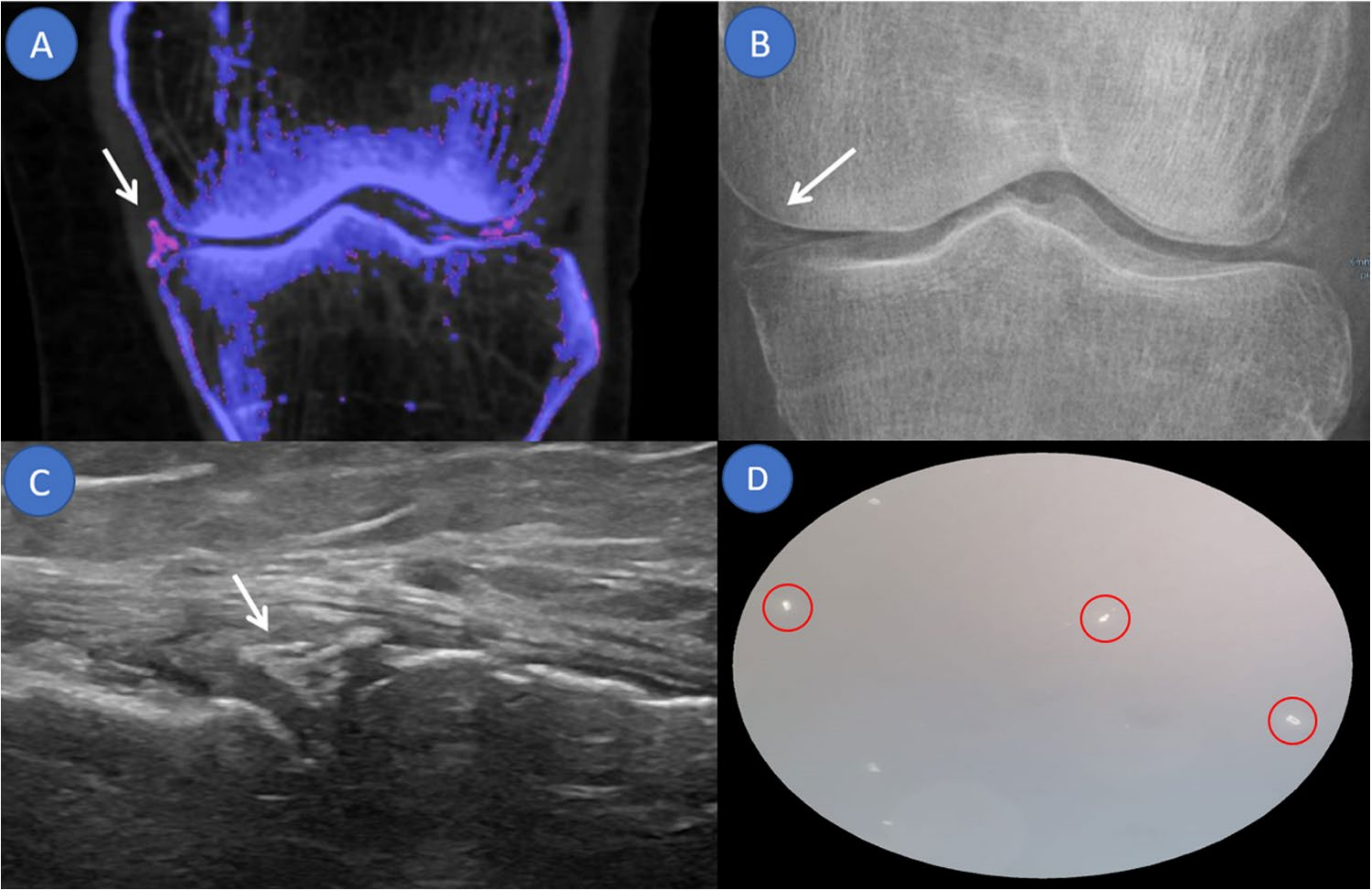
Overview of applied imaging modalities in an acute calcium pyrophosphate crystal arthritis of the left knee joint. The same calcium pyrophosphate deposition demonstrated along the hyaline cartilage of the medial and lateral menisci of the left knee. Prominent deposition at the lateral aspect of the medial meniscus (arrow) demonstrated utilizing different imaging modalities. **A** Dual energy computed tomography. **B** Conventional radiograph. **C** Ultrasound. **D** Compensated polarised light microscopy demonstrating rhomboid-shaped crystals indicative of calcium pyrophosphate disease

Twenty-four patients were diagnosed with acute gout flares (2 female, 22 male; mean age ± standard deviation [SD] 61 ± 12 years) and 11 with acute CPP crystal arthritis (4 female, 7 male; mean age ± SD, 70 ± 12 years). One patient tested negative for both via CPLM. Patient group allocation is shown in Fig. 1. A side-by-side DECT/MUS/CR/CPLM comparison of the same joint is demonstratively depicted in Fig. 2 for gout and Fig. 3 for CPP crystal arthritis. Out of 24 gout patients, 13 patients had a history of gout (54%), and one had a history of CPPD (9%). Two patients in the gout group had clinical tophaceous gout (8%) and 13 have experienced podagra (54%). Patient characteristics are summarised in Table 1. All patients underwent musculoskeletal ultrasound and most (32 out of 36) received radiographs at the time of arthrocentesis.

Altogether, 24 joints were positive for MSU deposits and 11 for CPP crystals in CPLM. The groups had a significant difference in disease history (*p* value: <0.001 Mann-Whitney *U* Test) with gout patients (*n* = 24) at a median of 20 months (IQR: 3–98) compared to 1 month (IQR: 0–2) in the CPP crystal arthritis cohort (*n* = 11). None of the analysed laboratory parameters differed between the groups. For example, serum urate demonstrated a Student’s *t* test *p* value of 0.11 (gout, mean 6.4 ± 1.9 mg/dL, CPP crystal arthritis, mean 5.3 ± 1.7 mg/dL), while CRP had a Mann-Whitney *U* test *p* value of 0.09 (median [IQR]; gout 10.9 mg/l [1.7–41]; CPPD 23.4 mg/l [10.7–119.6]).

**Table 1:** Patient characteristics

	**Acute gout flares (** ***n*** ** = 24)**	**Acute CPP crystal arthritis (** ***n*** ** = 11)**	**None (** ***n*** ** = 1)**
**Age, mean in years (SD)**	61 (12)	70 (12)	68
**Sex**			
Female, *n* (%)	2 (8)	4 (36)	0 (0)
Male, *n* (%)	22 (92)	7 (64)	1 (100)
**Symptom duration**			
0–6 months, *n* (%)	10 (42)	10 (91)^*^	1 (100)
7–60 months, *n* (%)	6 (25)	0 (0)	0 (0)
over 60 months, *n* (%)	8 (33)	0 (0)	0 (0)
**Symptom duration, median in months (IQR)**	20 (3–98)	0 (0–2)	0
History of gout/CPPD*			
Prior history, *n* (%)	13 (54)	1 (9)	1 (100)
New diagnosis, *n* (%)	8 (33)	10 (91)	0 (0)
**Latest serum urate, mean mg/dL (SD)**	6.4 (1.9)	5.3 (1.7)	4.8
**Highest serum urate, mean mg/dL (SD)**	8.1 (3.2)	7.2 (2.3)	4.8
**Podagra, ** ***n*** ** (%)**	13 (54)	1 (9)	1 (100)
**Affected joint that underwent DECT, ** ***n*** ** (%)**			
Hand joints (MCP/IP/PIP/DIP)	7 (29)	0 (0)	0 (0)
Wrist	0 (0)	4 (36)	1 (100)
Elbow	2 (8)	0 (0)	0 (0)
Shoulder	0 (0)	0 (0)	0 (0)
Hip	0 (0)	0 (0)	0 (0)
Knee	4 (17)	5 (46)	0 (0)
Ankle	1 (4)	1 (9)	0 (0)
Foot joints (MTP/IP/PIP/DIP)	10 (42)	1 (9)	0 (0)
**ACR/EULAR 2015 gout classification criteria score, median ** ***n*** ** (IQR)**	14.0 (10.0–16.0)	2.0 (1.0–3.5)	6.0 (0)
**Mono sodium urate load on DECT, median cm** ^**3**^ ** (IQR)**	0.17 (0–1.25)	0.01 (0–0.04)	0
**Serum calcium, mean mmol/l (SD)**	2.3 (0.1)	2.2 (0.3)	*
**Magnesium, mean mmol/l (SD)**	0.9 (0.2)	0.8 (0.1)	*
**Organic phosphate, mean mmol/l (SD)**	1.2 (0.2)	1.0 (0.1)	*
**Iron, mean µg/dl (SD)**	69.5 (39.5)	89.0 (43.3)	*
**Ferritin, mean ng/ml (SD)**	474.2 (387.5)	193.2 (201.1)	*
**CRP, mean mg/l (SD)**	26.8 (30.7)	66.4 (72.6)	1.5
**Leukocytes, mean ** ***n*** **/µl (SD)**	8.9 (3.9)	10.2 (3.4)	5.1
**Haemoglobin, mean g/dl (SD)**	12.7 (2.3)	11.7 (2.4)	14.0
**Thrombocytes, mean G/l (SD)**	262 (108)	342 (94)	179
**Thyroid stimulating hormone, mean µU/ml (SD)**	2.2 (1.7)	1.5 (0.9)	*

*CPP*, calcium pyrophosphate; *IQR*, inter quartile range; *DECT*, dual energy computed tomography; *MCP*, metacarpophalangeal joint; *IP*, interphalangeal joint; *PIP*, proximal interphalangeal joint; *DIP*, distal interphalangeal joint; *MTP*, metatarsophalangeal joint; *ACR,* American College of Rheumatology; *EULAR*, European League Against Rheumatism; *CRP*, C-reactive protein. ^*^Missing data

### Dual energy computed tomography

DECT imaging was able to correctly identify 15 out of 24 gout patients (63%) while nine were deemed false negatives resulting in a sensitivity of 63% (95% CI 0.41–0.81) and a specificity of 92% (95% CI 0.62–1.00). Six of the 11 CPP crystal arthritis patients (55%) were picked up on DECT with five false negatives, resulting in a sensitivity of 55% (95% CI 0.23–0.83) and a specificity of 92% (95% CI 0.74–0.99) for CPPD on DECT. Four of the six (67%) true positive CPP crystal arthritis cases on DECT had typical features on conventional radiographs. Inter user agreement between the radiologists (DK and CM) was good with an *α* of 0.873 using Krippendorff’s alpha.

The median disease duration of true positive gout cases on DECT was 43 months (IQR 5–103), while false negatives for gout (*n* = 9, 38%) had a median of 4 months (IQR 2–32). Patients with a positive gout finding on DECT were compared to the nine missed gout patients on DECT regarding serum urate, but no correlation could be established (Welch’s test *p* = 0.887, positive findings mean [± SD] 6.4 ± 1.7 mg/ml vs negative findings 6.3 ± 2.0 mg/ml).

**Fig. 4:**
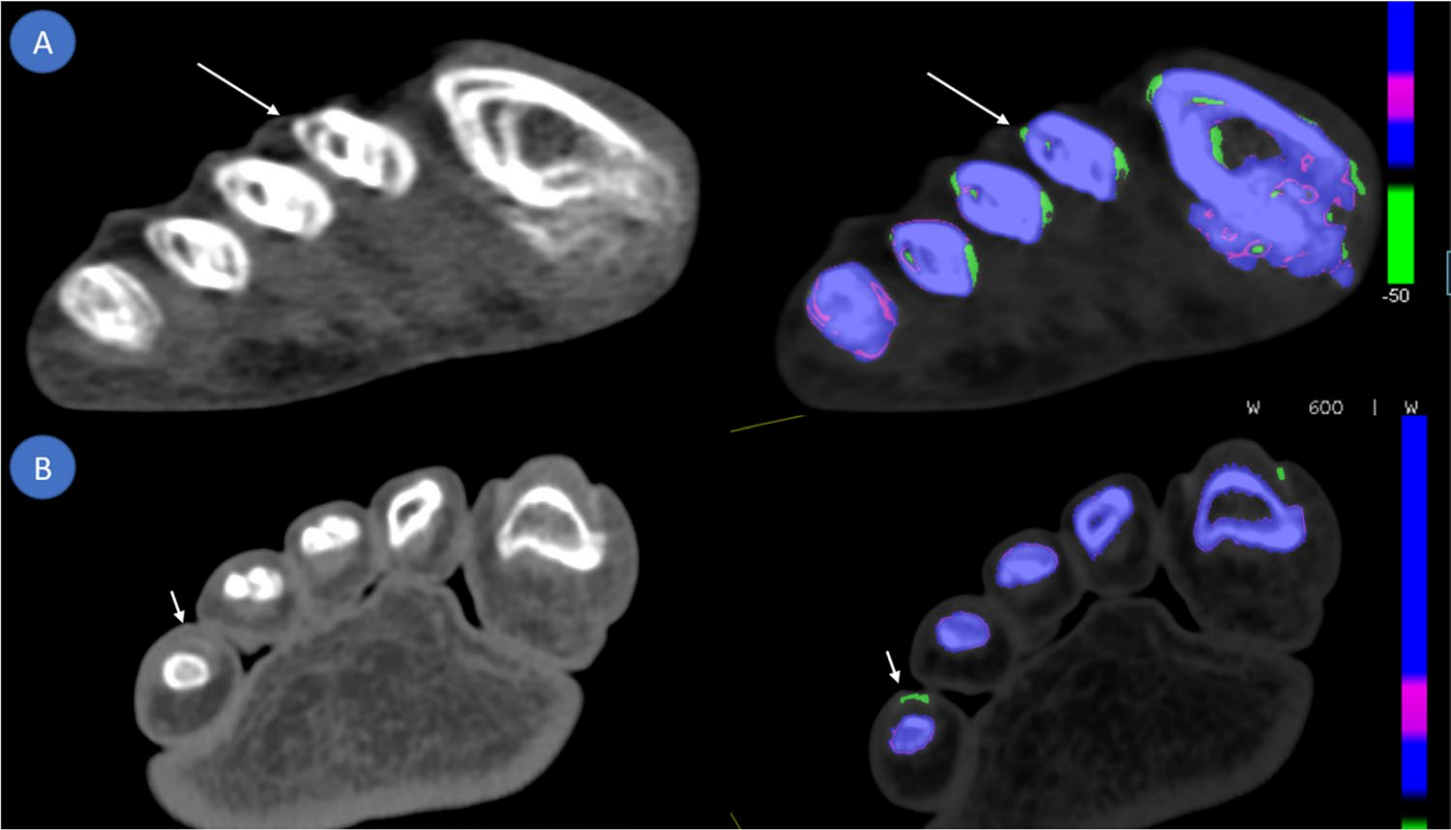
Artefacts observed on dual energy computed tomography scans of two patients. **A** Motion artefacts causing a false positive colour-coded DECT image. Non-colour-coded images acquired at 150 kV demonstrate streaking and blurring. The gout analysis software misinterprets these motion artefacts as MSU depositions (long arrows) on colour-coded images. **B** Nailbed artefacts (short arrows) causing another false positive result in another patient most likely due to similar dual energy indices of monosodium urate crystals and keratinous nails

Out of these nine false negatives, five (disease duration < 6 months) had a median disease duration of 2 months (IQR 1–3) while four (disease duration > 7 months) had a median disease duration of 82 months (IQR 26–295). Disease duration for CPP crystal arthritis was markedly shorter with a median of 1 month (IQR 0–2). One patient tested negative for both gout and CPP crystals. Two patients were classified as negative for either gout or acute CPP crystal arthritis due to artefacts while demonstrating positive colour-coded voxels on DECT images suggestive of gout. Both patients later turned out to be positive for gout via CPLM; their DECT images are illustrated in Fig. 4. The quality of the images was deemed sufficient to not be excluded from this study.

### Ultrasound

MUS achieved a sensitivity and specificity of 92% (95% CI 0.73–0.99) and 83% (95% CI 0.52–0.98) for gout, respectively. For acute CPP crystal arthritis, similar results were observed with a sensitivity of 91% (95% CI 0.59–1) and a specificity of 92% (95% CI 0.74–0.99).

### Conventional radiography

Conventional radiographs yielded the worst results with a sensitivity and specificity of 65% (95% CI 0.41–0.85) and 100% (95% CI 0.74–1.00) for gout, respectively. CPP crystals were less commonly detected with a sensitivity of 36% (95% CI 0.11–0.69) and a specificity of 90% (95% CI 0.70–0.99).

### Suspected clinical diagnosis

The suspected clinical diagnosis had comparable results to ultrasound with a sensitivity/specificity of 88% (95% CI 0.68–0.97)/75% (95% CI 0.43–0.95) for gout and 82% (95% CI 0.48–0.98)/88% (95% CI 0.69–0.97) for CPP crystal arthritis. The results of all three imaging modalities and the SCD are summarised in Table 2.

**Table 2:** Summary of the results for each imaging modality compared to compensated polarised light microscopy

Imaging modality\pathology and statistical results	Dual energy computed tomography *n* = 36	Musculoskeletal ultrasound *n* = 36	Conventional radiographs *n* = 32	Suspected clinical diagnosis *n* = 36
Acute gout flares				
Sensitivity (95% CI)	63% (0.41–0.81)	92% (0.73–0.99)	65% (0.41–0.85)	88% (0.68–0.97)
Specificity (95% CI)	92% (0.62–1.00)	83% (0.52–0.98)	100% (0.74–1.00)	75% (0.43–0.95)
PPV	94%	92%	100%	88%
NPV	55%	83%	63%	75%
Positive likelihood ratio	7.5	5.5	Inf	3.5
Negative likelihood ratio	0.4	0.1	0.35	0.167
Acute CPP crystal arthritis				
Sensitivity (95% CI)	55% (0.23–0.83)	91% (0.59–1.00)	36% (0.11–0.69)	82% (0.48–0.98)
Specificity (95% CI)	92% (0.74–0.99)	92% (0.74–0.99)	90% (0.70–0.99)	88% (0.69–0.97)
PPV	75%	83%	67%	75%
NPV	82%	96%	73%	92%
Positive likelihood ratio	6.818	11.364	3.818	6.818
Negative likelihood ratio	0.494	0.099	0.703	0.207

Sensitivities and specificities of examinations for acute gout flares and acute calcium pyrophosphate crystal arthritis (95% confidence intervals in brackets). *n* denotes the number of data sets for each modality. *PPV*, positive predictive value; *NPV*, negative predictive value

## Discussion

This is the first study to evaluate the diagnostic capability and yield of DECT to distinguish between acute gout flares and acute CPP crystal arthritis, comparing DECT results with standard imaging modalities such as ultrasound, conventional radiographs, suspected clinical diagnosis, and CPML in clinical practice. Invasive arthrocentesis with CPML still remains the gold standard for diagnosis of both diseases [8].

DECT has been validated [9, 20] as a diagnostic tool in suspected gout but seems to have a lower sensitivity than described in previous studies. Our observed sensitivity of 63% for DECT in gout was lower than other current diagnostic yield studies, compared to the 90% sensitivity described by Bongartz et al. [9] or 88% sensitivity described in a systematic review by Yu et al. [21]. A recent meta-analysis by Ogdie et al. found a pooled sensitivity of 87% and specificity of 84% [22]. A few factors might account for this discrepancy. Firstly, the number of examined gout patients in our study was lower than the 80 subjects analysed in the prospective study by Bongartz et al. [9] and the 750 by Yu et al. [21]. Secondly, out of the nine gout patients missed on DECT, five reported a disease duration of < 6 months with a median (IQR) disease duration of 2 months (1–3). Tophi and radiographic changes usually take many years to develop; on average, tophus formation occurs a decade after the first gout manifestation [23]. Other studies have also reported on the difficulties of diagnosing early gout manifestations via DECT [24]. MSU deposits in patients with a short disease duration might be at a stage of development where they are not detectable by DECT, which is limited to a spatial resolution of 0.25 mm [25], although lesions smaller than 1 mm can already be considered artefacts unless present at a typical gout localisation [26]. Similar results were found by Jia et al. [27] where the sensitivity of DECT for gout patients with initial onset was 35.7% and patients with a disease duration of less than 24 months yielded a sensitivity of 61.5%. A recent case report suggested that even large tophi are not picked up on the colour-coded DECT algorithm if they are not dense enough, requiring an approximate 15–20 volume percentage of urate [28]. The other four false negative gout patients on DECT had a median disease history (IQR) of 82 months (26–295) and have been receiving urate-lowering therapies. Mandell et al. [29] reported a mean time of tophi resolution of 10 months under pegloticase therapy, which is not available in Europe. More traditional therapies including febuxostat and allopurinol [30] lead to tophi resolution after 40 months of treatment. These four false negative gout patients on DECT were most likely not picked up on DECT due to long treatment time. Interestingly, the nine missed gout patients on DECT had a significantly lower ACR/EULAR 2015 gout classification score than the patients picked up on DECT (Student’s *t* test *p* = 0.03, 10.8 ± 3.9 vs 14.7 ± 4.1 points, respectively). Both ultrasound and DECT findings play into the score so that the difference cannot be explained alone on positive or negative DECT findings [18]. Although recent studies have found associations between positive gout findings on DECT with hyperuricemia [31], we were unable to reproduce such findings (Welch’s test *p* = 0.887, positive findings mean [± SD] 6.4 ± 1.7 mg/ml vs negative findings 6.3 ± 2.0 mg/ml) as urate values fluctuated even between the gout and CPP crystal arthritis groups (6.4 ± 1.9 vs 5.3 ± 1.7 mg/ml, *p* = 0.11).

Two false negative gout patients demonstrated positive colour-coded deposits on DECT images but were deemed as negatives due to motion artefacts and nailbed artefacts respectively and failed to demonstrate non-artefact related findings. Nailbed artefacts are most likely caused by a similar dual energy index value of keratin to that of MSU crystals [14] while motion artefacts are poorly understood and described as being confined to cortical bone [26].

DECT offered a sensitivity of 55% and a specificity of 92% for the detection of CPP crystals with a median disease history (IQR) of 1 month (0–2) using standardised scanning protocols for gout. To our knowledge, this was the first study comparing DECT scans to differentiate CPP crystals and MSU crystals and their effects of surrounding tissue using standardised gout protocols. Automatic volume quantification and colour coding for CPP crystals are not possible without modifying reconstruction parameters as DECT index values of calcium rich bone and calcium pyrophosphate deposits are similar and current gout pre-sets are not calibrated to differentiate these materials. The separation of CPP deposits from bone on DECT was recently demonstrated by Tedeschi et al. [32]. Using modified image reconstruction settings of existing gout protocols, they were able to colour code CPP deposits resulting in a sensitivity of 90–100%, compared to our finding of 55%. However, this was achieved at the expense of losing the ability to distinguish gout at the same time. Tanikawa et al. [33] were also able to colour-code ex vivo CPP deposits in meniscus specimens using modified reconstruction protocols reaching a sensitivity and specificity of 78% and 94% respectively. Current reconstruction protocols only allow colour coding of one variable, perhaps future protocols will allow coding for two variables with independent settings. All CPP crystal arthritis patients who demonstrated CPP typical depositions on radiographs also displayed CPP positive findings on DECT, in line with a previous study by Budzik et al., which also showed that DECT was not able to reliably identify CPP deposits in patients unless chondrocalcinosis was already visible on conventional X-rays [34].

Ultrasound sensitivity and specificity were 92% and 83% respectively for gout. When compared to a recent meta-analysis by Lee et al. [15], we were able to observe a higher sensitivity (92% vs 65%) albeit with a slightly lower specificity (83% vs 89%). MUS in diagnosis of CPP crystal arthritis has been validated by two meta analyses, Gamon et al. [35] and Filippou et al. [16], with pooled sensitivities of 34–77% and 88% respectively. Our own observed sensitivity of 92% for CPP depositions in MUS supports the results of Filippou et al. [16], a member of the OMERACT (Outcome Measures in Rheumatology) ultrasound subgroup.

Although both MUS and DECT have been validated for gout, DECT offers certain advantages over MUS. While MUS provides a cost-effective, non-ionising examination with good inter-user agreeability (mean inter-read *κ* values for the double contour sign = 0.93) [36], it remains at least partially user dependent [37]. DECT offers a unique objective way to quantify MSU deposits via automated volume assessment which is user independent and comparable across clinics and machines. This allows volume quantification follow-ups to monitor therapy success and provides comparability across clinics. Additionally, deep-seated MSU deposits which might be obscured by other anatomical structures on ultrasound are clearly visualised on DECT. No significant differences were observed between the groups regarding routinely acquired laboratory parameters. Current imaging techniques do have a major drawback compared to joint aspiration in acute arthritis, in that they cannot rule out bacterial infection. Thus, the need for joint aspiration remains in acute arthritis.

Our study encompasses a few limitations. Existing standardised scanning protocols which are currently optimised for gout, are not ideal for differentiating two calcium-rich substances such as normal bone and CPP depositions. Our study also suffered from a small patient pool, possibly skewing the results for the respective diagnostic modalities, especially musculoskeletal ultrasound. Further research with larger patient cohorts investigating specific CPP deposits and gout/CPP deposition hybrid protocols is needed to elucidate the role of DECT in CPP crystal arthritis.

## Conclusion

DECT has been validated for gout in a number of joints and offers an excellent tool for monitoring disease progression or therapy success but should not be used on its own for diagnostic purposes, as it can sometimes lead to false negatives in CPLM-positive acute gout flares. The role of DECT in acute CPP crystal arthritis remains unclear as the observed sensitivity is much lower than the already validated MUS using standardised scanning protocols. Current imaging techniques cannot replace joint aspiration in acute flares due to their inability to rule out septic arthritis. Further prospective studies, with larger patient cohorts and specialised DECT protocols, are needed to establish the role of DECT in diagnosing acute CPP crystal arthritis.

## Supplementary Information

Below is the link to the electronic supplementary material.

Supplementary file1 (DOCX 41 KB)

## Data Availability

Raw date can be provided upon request.
