# Supplementary material for: The role of dual energy computed tomography in the differentiation of acute gout flares and acute calcium pyrophosphate crystal arthritis
Source: Clin Rheumatol. 2021 Oct 9;41(1):223–33. doi: 10.1007/s10067-021-05949-4 (PMC8724058; doi:10.1007/s10067-021-05949-4)
Supplement: Supplementary file 1 — Supplementary file1 (DOCX 41 KB) [file 10067_2021_5949_MOESM1_ESM.docx]

Supplementary Table S1
Dual energy computed tomography scan parameters

| Scan parameters |  |
| --- | --- |
| Scan type | Spiral |
| Collimation, mm | 0.6 |
| Slice thickness, mm | 0.75 |
| Pitch | 0.7 |
| kVp |  |
| Tube A | 80 |
| Tube B | 150 |
| mAs |  |
| Tube A (variable dose modulation) | Mean (SD): 111.4 (10.3); Range: 94 - 139 |
| Tube B (variable dose modulation) | Mean (SD): 75.1 (6.6); Range: 66 - 95 |
| CTDI, mGy | Variable |
| Reconstruction |  |
| Kernel | Qr40d\2 |
| Dual energy gout settings |  |
| Soft tissue (80 kV) | 50 |
| Soft tissue (150 kV) | 50 |
| Resolution | 4 |
| Dual energy ratio | 1.4 |
| Min. HU | 150 |
| Max. HU | 500 |
| Range | Air distance 5, bone distance 10 |

kVp: kilovoltage peak; mGy: milligray; kV: kilovolt; HU: Hounsfield units.
